# Isolation of *Bacillus amyloliquefaciens* D39 and Identification of Its Antimicrobial Proteins Active Against Chestnut Blight

**DOI:** 10.3390/microorganisms13061302

**Published:** 2025-06-03

**Authors:** Tingting Deng, Linmin Wang, Tianhui Zhu

**Affiliations:** 1 Department of Forest Protection, College of Forestry, Sichuan Agricultural University, Chengdu 611130, China; deng_tingting001@163.com; 2 School of Agronomy and Horticulture, Chengdu Agricultural College, Chengdu 611130, China

**Keywords:** chestnut blight, *Bacillus amyloliquefaciens*, biological control, protein purification, protein structure

## Abstract

Chestnut blight, caused by *Cryphonectria parasitica* (Murrill) M.E. Bar, is a destructive fungal disease threatening chestnut cultivation and production. In response to the limitations of chemical control, biological control using antagonistic microbes has gained increasing attention. A rhizosphere-derived bacterium, strain D39, was isolated from healthy chestnut trees and identified as *Bacillus amyloliquefaciens* based on morphological characteristics and the phylogenetic analysis of 16S rRNA and *gyrA* genes. The antifungal activity of strain D39 against *C. parasitica* was evaluated using dual-culture and double-layer Oxford cup assays. The strain exhibited broad-spectrum and stable antagonistic effects and harbored five key genes associated with antimicrobial compound biosynthesis (*srfAA*, *ituC*, *fenD*, *bmyB*, and *bacA*), as confirmed by PCR. A 145 kDa extracellular protein with strong antifungal activity was extracted and purified by ammonium sulfate precipitation, DEAE ion-exchange chromatography, and Sephadex G-75 gel filtration. LC-MS analysis identified the protein as a serine peptidase belonging to the S8 family, and its structure was predicted using multiple bioinformatic tools. In pot experiments, treatment with the strain D39 significantly reduced disease severity, achieving control efficiencies of 66.07% and 70.89% at 10 and 20 days post-inoculation, respectively. These results demonstrate that *B. amyloliquefaciens* D39 has strong potential as a biocontrol agent against chestnut blight, offering an effective and environmentally friendly alternative for disease management.

## 1. Introduction

Chestnut, belonging to the family Fagaceae, is a plant with high nutritional value and strong tolerance to adverse conditions, making it an economically important forest tree species for nuts [1,2]. However, chestnut blight, caused by the fungus *Cryphonectria parasitica* (Murrill) M.E. Bar, is catastrophic for chestnut trees, resulting in substantial economic losses [3]. This disease typically manifests as sunken cankers on the bark, wilting foliage, necrotic lesions, and eventual girdling of the stem, which can rapidly kill branches and entire trees [4]. Chemical pesticides can be used to control chestnut blight [5,6], but extensive use of these chemicals may lead to the development of resistance to pests, environmental pollution, and potential harm to human health [7,8]. In contrast, biological control has emerged as a promising and environmentally friendly strategy for managing plant fungal diseases, including chestnut blight. It relies on the use of beneficial microorganisms, such as bacteria, fungi, or viruses, to suppress plant pathogens through mechanisms like competition for nutrients and space, antibiosis, parasitism, and the induction of systemic resistance in the host plant [9,10].

Notably, hypovirulent mycoviruses such as *Cryphonectria hypovirus* (CHV) have demonstrated considerable potential in the biological control of chestnut blight caused by *C parasitica* [11]. Although CHV has been extensively studied and successfully applied in various regions [12,13,14], its effectiveness has exhibited considerable regional variability in areas such as Spain and certain parts of the United States [15,16,17,18]. In China, although various types of CHV have been isolated and characterized, their practical application in chestnut blight control remains limited [19,20]. This lack of research may be attributed to the complex chestnut population system in China, with a high diversity of hypoviruses and vegetative compatibility groups. The complexity and diversity increase the difficulty of using hypovirulent strains for the control of chestnut blight [12,21,22,23,24]. Therefore, using hypovirulent strains for biological control remains challenging in China.

Among various biocontrol agents, *Bacillus* spp. have gained considerable attention due to their safety, adaptability, and effectiveness [25]. These bacteria are ubiquitous in nature, including soils, rhizosphere, and as endophytes, and have been widely studied for their antifungal properties. They can form spores, which allows them to survive in nutrient-deficient settings and extreme environments such as high temperatures, drought, and freezing conditions [26,27]. Additionally, species of the genus *Bacillus* reproduce rapidly and combat pathogens through various mechanisms, including competition, the production of antagonistic secondary metabolites (such as surfactin, iturin, and fengycin), the induction of systemic resistance in plants, enzymatic lysis, and the promotion of plant growth [28,29,30,31]. Recent studies have highlighted the pivotal role of soil-derived *Bacillus* spp. in managing fungal plant diseases through multiple mechanisms, including the production of lipopeptides, cell wall–degrading enzymes, siderophores, and biofilm formation, as well as the induction of systemic resistance in host plants [32,33]. These mechanisms enhance the ability of *Bacillus* strains to colonize the rhizosphere and outcompete phytopathogens in complex soil environments.

To address the limitations of existing biocontrol strategies and the practical challenges of applying hypovirulent strains in China, this study presents a novel approach by isolating and characterizing a new strain of *Bacillus amyloliquefaciens* (D39) from the rhizosphere of healthy chestnut trees. This strain exhibited significant and stable antagonistic activity against *C. parasitica,* as confirmed by in vitro dual-culture assays and pot experiments. Unlike previous studies focusing on known *Bacillus* strains or hypovirulent mycoviruses, this work further purified and identified a previously unreported extracellular antifungal protein—a serine peptidase, belonging to the S8 family—with potent antifungal activity. The structure of this protein was predicted using bioinformatic tools. By integrating microbial isolation, protein purification, structural prediction, and efficacy validation, this study provides new insights into the mechanisms of *Bacillus*-based biocontrol and lays a solid theoretical and practical foundation for the development of effective and environmentally friendly formulations against chestnut blight.

## 2. Materials and Methods

### 2.1. Bacterial Strains, Soil Samples, Chestnut Seedlings, and Culture Medium

The fungal strain C. parasitica (GenBank KJ528273) and ten additional plant pathogenic fungi—namely Fusarium solani, Fusarium tricinctum, Phomopsis capsici, Fusarium fujikuroi, Arthrinium phaeospermum, Fusarium incarnatum-equiseti species complex, Botryosphaeria dothidea, Arthrinium rasikravindrii, Fusarium oxysporum, and Neofusicoccum parvum—were all obtained from the Department of Forest Protection, College of Forestry, Sichuan Agricultural University. All fungal strains were previously isolated, identified, and preserved by the same department, as described in references [34,35]. Rhizosphere soils from healthy chestnut trees in Sichuan Agricultural University chestnut orchard (30°33′27′′ N, 103°39′5′′ E) were mixed and stored at 4 °C. The soil at the site is classified as sandy loam with a slightly acidic pH ranging from 5.9 to 6.3. The chestnut seedlings were of the three-year-old Dahongpao variety, with an average height of 60–70 cm. Lysogeny Broth (LB) medium and potato dextrose agar (PDA) medium were used for bacterial and fungal cultivation, respectively. The LB medium was composed of 10 g tryptone, 5 g yeast extract, and 10 g NaCl per liter of distilled water, adjusted to pH 7.0. The PDA medium was prepared by boiling 200 g of peeled potatoes in 1 L of distilled water, filtering the extract, and adding 20 g glucose and 20 g agar, followed by pH adjustment to 7.0. The media formulations were prepared based on reference [36], with appropriate modifications.

### 2.2. Isolation and Identification of Antagonistic Bacteria

#### 2.2.1. Isolation, Purification, and Preservation of *Bacillus* spp.

Soil samples (10 g) were mixed with 90 mL sterile water and heated in a water bath at 80 °C for 20 min to eliminate vegetative bacteria [37]. After serial dilution (10^−3^ to 10^−7^ g/mL), 100 μL aliquots from each dilution were plated on LB agar and incubated at 28 °C for 24–48 h (triplicate plates per dilution). Subsequently, colonies were initially classified based on morphology and color. Representative single colonies were isolated by quadrant streaking and subsequently preserved in 30% glycerol at −80 °C for long-term storage.

#### 2.2.2. Screening of Antagonistic Bacteria

Antagonistic bacteria were primarily screened via a four-point mycelial plug assay [38], where *C. parasitica* mycelial plugs (Φ = 5 mm) were centrally positioned on PDA plates with test strains inoculated perpendicularly at 2.5 cm intervals. Each treatment was set up with three replicates. Plates inoculated only with *C. parasitica* mycelial plugs were used as the control. Following incubation at 28 °C until control colonies achieved full plate coverage, mycelial diameters were measured in two perpendicular directions to calculate inhibition zone widths and rates (Equations (1) and (2)), with strains demonstrating significant inhibitory capacity being advanced for further analysis.

Secondary screening was conducted using the Oxford cup method [39]: spore-embedded PDA plates were prepared by homogenizing *C. parasitica* spore suspension (1 × 10^8^ CFU/mL, 5% v/v) with sterilized PDA medium cooled to 50 °C, poured into plates (20 mL/plate), and solidified. Two Oxford cups were implanted per plate and loaded with 200 μL filter-sterilized fermentation broth prepared from antagonistic bacterial isolates (each treatment performed in triplicate; sterile water served as the control). Optimal strains were selected based on inhibition zone diameters measured in two perpendicular directions following 5-day incubation at 28 °C.

(1) Inhibition zone width=R1−R2

(2) $$\mathrm{Inhibition}\;\mathrm{rate}=\frac{R3-R4}{R3}$$

where R1 is the diameter of the inhibition zone; R2 is the diameter of the antagonistic bacterial colony; R3 is the diameter of the control; and R4 is the diameter of antagonist treatment.

#### 2.2.3. Identification of Antagonistic Bacteria

Bacterial identification integrated colony morphology with physiological–biochemical profiling (Gram-staining, spore staining, starch hydrolysis, etc.) [40]. Genomic DNA was extracted from the bacterial strains using a bacterial genomic DNA extraction kit (TianGen, Beijing, China). The 16S rRNA gene sequence was then amplified using the universal bacterial primers 27F and 1492R [41]. The PCR mixture was denatured for 5 min at 95 °C, followed by 30 cycles of 60 s at 95 °C, 30 s at 55 °C, and 1.5 min at 72 °C, and finally one cycle of 7 min at 72 °C. The *gyrA* gene sequence was amplified using the specific primers gyrA-F and gyrA-R [25]. This was followed by PCR cycling under the following denaturation conditions: 2 min at 94 °C, 35 cycles of 30 s at 94 °C, 30 s at 50 °C, and 1 min at 72 °C, and finally one cycle of 1 min at 72 °C. The products were examined by agarose gel electrophoresis and subsequently sequenced using the Sanger sequencing platform ABI 3730XL DNA Analyzer (Applied Biosystems, Foster City, CA, USA). The resulting sequences were compared and analyzed using the National Center for Biotechnology Information (NCBI) BLAST database (https://www.ncbi.nlm.nih.gov/, accessed on 5 September 2021). Bacterial strains with a high sequence identity (>95%) to the target sequence were selected and downloaded. Multiple sequence alignment was performed using MEGA7.0, and a phylogenetic tree was reconstructed using Bayesian methods with PhyloSuite version 1.2.2 [42]. Bayesian inference phylogenies were inferred using MrBayes 3.2.6 [43] under a partition model (two parallel runs, 2,000,000 generations), with the initial 25% of the sampled data discarded as burn-in. The results were visualized and edited using FigTree version 1.4.4 (http://tree.bio.ed.ac.uk/software/figtree, accessed on 12 February 2022).

### 2.3. Analysis of the Antimicrobial Capability of Strain D39

#### 2.3.1. Subculture Cultivation

Strain D39 was inoculated onto LB solid medium and subcultured for 10 generations, with each generation cultured for 24 h. *C. parasitica* was used as an indicator organism. The four-spot method with fungal cakes was employed to measure the antimicrobial activity of each generation of strain D39. A plate without antagonistic bacteria served as the control. After the control plate was fully colonized, the inhibition rate was calculated using Equation (2).

#### 2.3.2. Growth Curve and Antimicrobial Capability

Sixteen bottles of strain D39 fermentation liquid were cultured in a shaking incubator, with each bottle containing 100 mL. One bottle was taken every 6 h to measure the optical density at the wavelength of 600 nm (OD_600_). OD_600_ values of the uninoculated culture liquid served as a control, with a total of 16 bottles measured. The microbial growth curve was plotted, with the fermentation time on the horizontal axis and the OD_600_ values of the fermentation liquid on the vertical axis. Moreover, the antimicrobial activity of the sterile fermentation liquid from each bottle against the chestnut blight pathogen was determined. Briefly, the bacterial liquid was made into sterile fermentation liquid and 200 μL was spread on the surface of a PDA plate, with the central part of the plate inoculated with a mycelial (φ = 5 mm) plug of *C. parasitica* in triplicate. A PDA plate with only the pathogen cake served as a control. After the control plate was fully colonized, the inhibition rate was calculated according to Equation (2). An antimicrobial activity curve was plotted—with time on the horizontal axis and the inhibition rate on the vertical axis—and compared with the growth curve.

#### 2.3.3. Antimicrobial Spectrum

Fungal mycelial plugs (φ = 5 mm) of the 10 plant pathogenic fungi listed in Section 2.1 were cut from the actively growing margins of colonies on PDA plates and placed in the center of fresh PDA plates. Antagonistic bacterial strains were inoculated at four equidistant points, each 2.5 cm from the fungal plug in perpendicular directions. Sterile water was used as a negative control. All plates were incubated at a constant temperature of 28 °C for 4 days. The inhibition rate was calculated according to Equation (2) to evaluate the inhibitory effect of the antagonistic bacterial strains.

#### 2.3.4. PCR detection of Biocontrol-Related Genes in Strain D39

Research has demonstrated a significant correlation between the biological control of plant pathogens and the biosynthetic genes of antimicrobial peptides (AMPs), including *srfAA* (surfactin), *bmyB* (bacillomycin), *bacA* (bacilysin), *fenD* (fengycin) and *ituC* (iturin) [44]. Given this established relationship, the antibacterial potential of strain D39 was investigated through the detection of these AMP biosynthetic genes. To this end, polymerase chain reaction (PCR) was employed to amplify the corresponding gene loci using specific primers targeting *srfAA*, *bmyB*, *bacA*, *fenD*, and *ituC* (Table S1). The thermal cycling conditions were 4 min at 95 °C, followed by 34 cycles of 40 s at 95 °C, 40 s at 55 °C, 1.5 min at 72 °C, and then a final extension of 10 min at 72 °C. PCR products were analyzed by agarose gel electrophoresis. A 5 μL sample of the PCR products was loaded onto a 1.0% agarose gel in 1X TBE buffer and separated in a horizontal electrophoresis chamber at 110 V and 160 mA for 25 min. The validated amplicons were then sequenced via Sanger sequencing technology using an ABI 3730XL DNA Analyzer (Applied Biosystems, USA). Sequencing results were then compared and analyzed using NCBI Nucleotide BLAST.

#### 2.3.5. Pot Experiments

Pot control experiments were conducted in the forest protection experimental greenhouse at Sichuan Agricultural University. First, an activated *B. amyloliquefaciens* D39 strain was transferred into LB medium using a sterilized loop and incubated at 28 °C for 24 h to obtain the fermentation broth (1 × 10^6^ CFU/mL). Healthy chestnut seedlings were selected and their main stems were punctured with a sterilized needle to create wounds penetrating to the xylem, with a total of three wounds per branch. Subsequently, a fungal mycelial plug of *C. parasitica* (φ = 10 mm) was attached to the wounds and moisture was maintained. After disease symptoms appeared on the chestnut seedlings, plants with uniform growth were selected for the control experiment to verify the biocontrol effect of the fermentation broth of strain D39 against *C. parasitica*.

The pot experiment was set up with two treatment groups, each with three replicates. Each replicate contained 10 chestnut seedlings, totaling 60 plants. For treatment A, a sterile gauze was soaked in the fermentation broth of antagonistic bacteria, wrapped around the wounds of the chestnut seedlings, and tightly sealed with plastic wrap to maintain moisture. Treatment B, the control group, was treated with sterile water in the same manner.

After treatment with the bacterial fermentation broth, chestnut seedlings were assessed for disease severity on days 10 and 20, and the incidence rate, disease index, and control effect were calculated according to Equations (3)–(5) [45]. Disease severity was categorized as follows: L0, the plant is healthy with no visible signs or symptoms of disease; L1, the horizontal diameter of the canker wound accounts for less than 1/4 of the trunk circumference; L2, 1/4–1/2 of the trunk circumference is affected; L3, 1/2–3/4 of the trunk circumference is affected; and L4, the plant is dead.

(3) $$\mathrm{Incidence}\;\mathrm{rate}\left(\%\right)=\frac{\mathrm{Number}\;\mathrm{of}\;\mathrm{affected}\;\mathrm{sites}}{\mathrm{Total}\;\mathrm{number}\;\mathrm{of}\;\mathrm{inoculation}\;\mathrm{sites}}\times 100\%,\;$$

(4) $$\mathrm{Disease}\;\mathrm{index}=\frac{\sum \left(\mathrm{diseased}\;\mathrm{plant}\;\mathrm{numbers}\;\times \;\mathrm{the}\;\mathrm{levels}\;\mathrm{of}\;\mathrm{severity}\right)}{\left(\mathrm{total}\;\mathrm{number}\;\mathrm{of}\;\mathrm{investigations}\;\times \;\mathrm{the}\;\mathrm{highest}\;\mathrm{level}\;\mathrm{of}\;\mathrm{severity}\right)}\times 100,\;$$

(5) $$\mathrm{Control}\;\mathrm{effect}\;\left(\%\right)=\frac{\mathrm{Control}\;\mathrm{disease}\;\mathrm{index}-\mathrm{Treatment}\;\mathrm{disease}\;\mathrm{index}}{\mathrm{Control}\;\mathrm{disease}\;\mathrm{index}}\times 100\%.$$

### 2.4. Extraction and Purification of Antimicrobial Substances

#### 2.4.1. Ammonium Sulfate Precipitation for Preliminary Extraction of Antimicrobial Substances

Ammonium sulfate ((NH_4_)_2_SO_4_) powder was added to six portions of 100 mL of sterile fermentation broth of strain D39 to achieve saturation levels of 0%, 20%, 40%, 60%, 80%, and 100% (at 0 °C) and then left to stand overnight at 4 °C. After centrifugation at 4000 rpm for 30 min, the supernatant and precipitate were separated. The precipitate was dissolved in a double volume of 50 mmol/L phosphate-buffered saline (PBS) solution (pH 7.2). Both the supernatant and the redissolved precipitate were dialyzed in PBS at 4 °C for 24 h to remove salts and concentrated to 7 mL to obtain a crude antimicrobial extract.

The antimicrobial activity of the supernatant and precipitate against the chestnut blight pathogen was evaluated using the double-layer Oxford cup method. First, PDA was poured into Petri dishes at a depth of approximately 5 mm, and after solidification, sterilized Oxford cups were placed on the plates, ensuring that they did not interfere with each other’s positions. A spore suspension of *C. parasitica* (1 × 10^8^ CFU/mL) was then added and cooled (to 50 °C) PDA medium at a ratio of 5% (v/v), mixed evenly, and then poured into the plates to form double-layer PDA plates. After the plates were cooled, 200 μL of the supernatant or the redissolved precipitate was added to the Oxford cups, with 50 mmol/L PBS serving as the control group, and each treatment was repeated three times.

#### 2.4.2. Separation and Purification of Antimicrobial Proteins and Determination of Protein

The crude antimicrobial extract (2 mL) was loaded onto a column packed with DEAE Sepharose Fast Flow (15 mm × 200 mm, Solarbio, Beijing, China) that had been pre-equilibrated with 50 mM Tris-HCl buffer (pH 7.5). Cation exchange chromatography was initiated with the same buffer, followed by stepwise elution using a sodium chloride gradient with increasing concentrations of 0, 0.6, 0.8, and 1.0 M.

After preliminary purification, protein peaks with antimicrobial activity were selected and further purified using Sephadex G-75 (15 mm × 500 mm, Solarbio, Beijing, China). The column was pre-washed and equilibrated with 50 mM Tris-HCl buffer (pH 7.5) before sample application. Elution was performed using the same buffer to maintain consistent conditions throughout the purification process.

Both purification steps were conducted at a flow rate of 2 mL/min, with the protein solution collected at one-minute intervals. Protein peaks were pooled based on the chromatogram. The pooled fractions from the preliminary purification were dialyzed in 50 mM Tris-HCl buffer (pH 7.5) at 4 °C for 24 h to remove salts. The pooled fractions from both purification steps were concentrated to 2 mL. Their effectiveness was evaluated using the double-layer Oxford cup method (as described in Section 2.4.1), with 50 mM Tris-HCl buffer (pH 7.5) serving as the negative control group, and each treatment was repeated three times. Protein concentration was measured according to the Bradford method [46]. The molecular weight of the protein was calculated using sodium dodecyl sulfate-polyacrylamide gel electrophoresis (SDS–PAGE) [47].

#### 2.4.3. RPLC-MS and Data Analysis

The antimicrobial protein was digested with trypsin (V5280, Promega, Madison, WI, USA), and the resulting peptides were desalted using Ziptip C18 tips and vacuum-dried. The peptides were redissolved in 0.1% formic acid, vortexed thoroughly, and centrifuged at 13,500 rpm for 20 min at 4 °C. The supernatant (10 μL) was analyzed by reverse-phase liquid chromatography–mass spectrometry (RPLC-MS) using a Thermo EASY nLC system coupled with a Thermo Scientific QE HF mass spectrometer (both from Thermo, Waltham, MA, USA). Chromatographic separation was performed on a 75 μm i.d. × 150 mm Acclaim PepMap RSLC C18 column packed with a 2 μm particle size, 100 Å pore size, and featuring a nanoViper design (Thermo Fisher Scientific, Waltham, MA, USA). The mobile phases consisted of 0.1% formic acid in water (mobile phase A) and 0.1% formic acid in 80% acetonitrile (mobile phase B). Details of liquid chromatography and mass spectrometry parameters are provided in Tables S2 and S3.

The MS data were searched against the UniProt proteome database (UP000502253, Nov 11, 2021) using PEAKS software (version 8.0; Bioinformatics Solutions Inc., Toronto, Canada). Details of the database search parameters are provided in Table S4.

#### 2.4.4. Protein Structure Prediction

The primary structure of the antimicrobial protein was analyzed using ExPASy (http://web.expasy.org/protparam/, accessed on 23 February 2022) [48], secondary structure prediction was performed using PSIPRED (http://bioinf.cs.ucl.ac.uk/psipred/, accessed on 23 February 2022) [49], and tertiary structure modeling was conducted using Phyre2 (http://www.sbg.bio.ic.ac.uk/phyre2/html/page.cgi?id=index, accessed on 23 February 2022) [50], trRosetta (https://yanglab.qd.sdu.edu.cn/trRosetta/, accessed on 17 March 2022) [51], and I-TASSER (https://zhanggroup.org/I-TASSER/, accessed on 3 March 2022) [52].

Phyre2 and I-TASSER use homology modeling, whereas trRosetta integrates deep learning technology with homologous templates for tertiary structure predictions. The accuracy of the tertiary structures predicted by different methods was compared using the TM-score value (https://zhanggroup.org/TM-score/, accessed on 18 March 2022), which assesses the topological similarity of protein structures. A TM-score between 0.5 and 1.0 typically indicates a correct topology, while a TM-score less than 0.17 suggests random structural similarity [53].

### 2.5. Statistical Analysis

All experiments were conducted in triplicate unless otherwise stated. Data are expressed as mean ± standard deviation (SD). One-way analysis of variance (ANOVA) was performed using SPSS 2019 (IBM, Armonk, NY, USA), and significant differences among treatment means were determined by the Waller–Duncan post hoc test at a significance level of *p* < 0.05. Excel 2019 (Microsoft, Redmond, WA, USA) was used for data tabulation and graphing. In figures and tables, “NS” denotes non-significant differences (*p* > 0.05), “*” denotes significant differences (*p* < 0.05), and different lowercase letters indicate statistically significant differences between groups as determined by the Waller–Duncan test.

## 3. Results

### 3.1. Isolation of Antagonistic Strain D39

**Figure 1:**
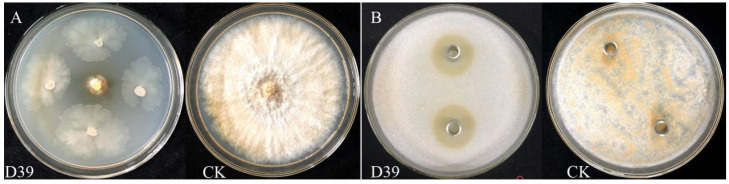
Primary and secondary screening effects of strain D39. (**A**) A mycelial plug of *C. parasitica* was placed at the center of a PDA plate, and the strain D39 was inoculated at 2.50 cm from the disk in a direction perpendicular to it; CK: a 5 mm mycelial plug of *C. parasitica*, was used as the blank control. (**B**) *C. parasitica* spore suspension was added to PDA and thoroughly mixed. Then, 200 μL of sterile fermentation broth from strain D39 was placed into Oxford cups containing distilled water serving as the blank control.

A total of 89 bacterial strains were isolated from the soil according to colony morphology. After the primary screening, 14 strains exhibited varying degrees of antagonistic activity. During the secondary screening, five strains—D22, D39, D40, D42, and D45—formed inhibition zones on the PDA plates containing mycelial plug of *C. parasitica*. Among the five strains, D39 showed the strongest antimicrobial activity, with an inhibition zone diameter of 2.39 ± 0.07 cm (Table 1 and Figure 1). Therefore, strain D39 was selected as the antagonistic bacterium against *C. parasitica* for further analysis.

### 3.2. Identification of Strain D39 as B. amyloliquefaciens

Strain D39 formed opaque, milky-white colonies on LB agar. The colonies were circular with irregular edges and a raised center (Figure S1A,B). Strain D39 was identified as a Gram–positive, spore-forming bacterium. The strain demonstrated proficiency in hydrolyzing starch and reducing nitrate, producing positive results in the gelatin liquefaction, Voges–Proskauer reaction, and catalase tests; however, negative results were observed in the methyl red test, phenylalanine deaminase test, and citrate utilization test (Table 2 and Figure S1C–L). In accordance with its morphological characteristics and physiological and biochemical properties, strain D39 was preliminarily identified as *Bacillus* spp.

**Figure 2:**
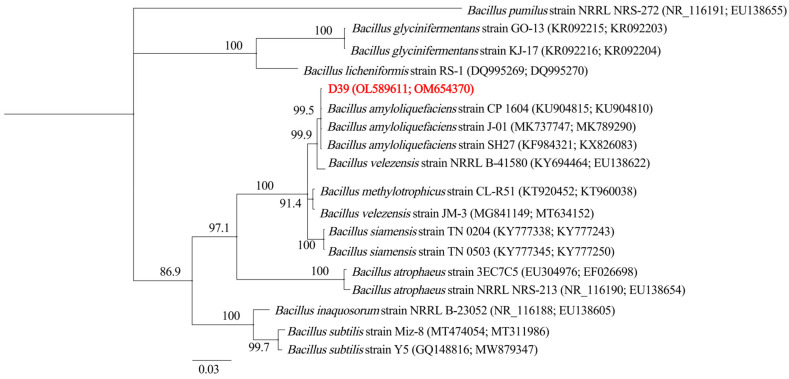
Phylogenetic tree inferred from the combined 16S rRNA gene and *gyrA* sequence alignment. The Bayesian consensus tree was constructed over 2 million generations, with the initial 25% of data discarded as burn-in. Support for each branch in the tree was evaluated using posterior probability values, which are indicated on the branches. The scale bar indicates 0.03 expected changes per site. The Latin names and Clade numbers are provided on the right of the tree. NCBI accession numbers for the 16S rRNA gene and *gyrA* sequences of each strain are shown in parentheses. The tree is rooted with *Bacillus pumilus* (strain NRRL NRS-272). Strain D39 is highlighted in bold and red.

The 16S rRNA gene (1039 bp) and *gyrA* locus (969 bp) of strain D39 were successfully amplified and sequenced (GenBank accession numbers OL589611 and OM654370, respectively). Phylogenetic analysis of concatenated sequences from these genetic markers demonstrated that D39 formed a monophyletic clade with *B. amyloliquefaciens* strains (Figure 2). Combined with morphological observations and biochemical profiling, this polyphasic taxonomic approach definitively classifies strain D39 as *B. amyloliquefaciens*.

### 3.3. Antimicrobial Activity of Strain D39

#### 3.3.1. Subculturing

After 10 consecutive subcultures, the inhibition rate of strain D39 showed no significant change (*p* > 0.05), indicating that the antimicrobial activity of the strain was stable. Therefore, the strain could be used as an antagonist against *C. parasitica* in further studies.

#### 3.3.2. Growth Curve and Antimicrobial Activity

**Figure 3:**
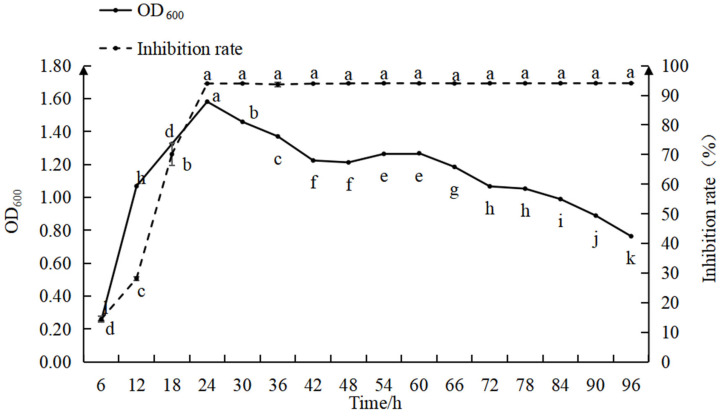
Relationship between the inhibition rate and growth curve for strain D39. Different lowercase letters denote significant differences among treatments (Waller–Duncan test, *p* < 0.05).

The relationship between the biomass and antimicrobial activity of strain D39 is shown in Figure 3. During 0–12 h, the bacterial count increased rapidly, while the inhibition efficiency gradually improved. Between 12 and 24 h, the bacterial count increased slowly, while the inhibition rate rose quickly. At 24 h, the bacterial count and inhibition rate reached their maximum values, with the inhibition rate approaching 100% and optimal antimicrobial activity was maintained in the subsequent period. From 24 to 42 h, the bacterial count gradually declined, stabilized between 42 and 66 h, and then decreased again from 66 to 96 h. The findings indicate that with a 5% inoculation rate, both the bacterial count and inhibition rate peaked at 24 h, after which the bacterial count began to decline while the inhibition rate remained relatively constant.

During the 0–12 h period, the bacterial count increased rapidly and the nutrients in the culture medium were primarily used for the increase in bacterial numbers, resulting in the low production of antimicrobial substances and, consequently, a low inhibition rate (*p* < 0.05). From 12 to 24 h, as as bacterial growth slowed, nutrients were increasingly directed toward the synthesis of antimicrobial substances, leading to a rapid increase in the inhibition rate. When the bacterial count reached its peak, the production of antimicrobial substances accelerated, bringing the inhibition rate close to 100%. During the 24–96 h period, though the bacterial count gradually decreased, the culture medium still contained a high concentration of antimicrobial substances, allowing the inhibition rate to remain at its maximum of 100%. These observations suggest that prior to peak bacterial density, the antimicrobial effect is influenced by both the number of antagonistic bacteria and the concentration of antimicrobial substances; after the bacterial population peaks, the antimicrobial effect is primarily dependent on the concentration of antimicrobial substances.

#### 3.3.3. Broad-Spectrum Antifungal Activity of Strain D39

**Figure 4:**
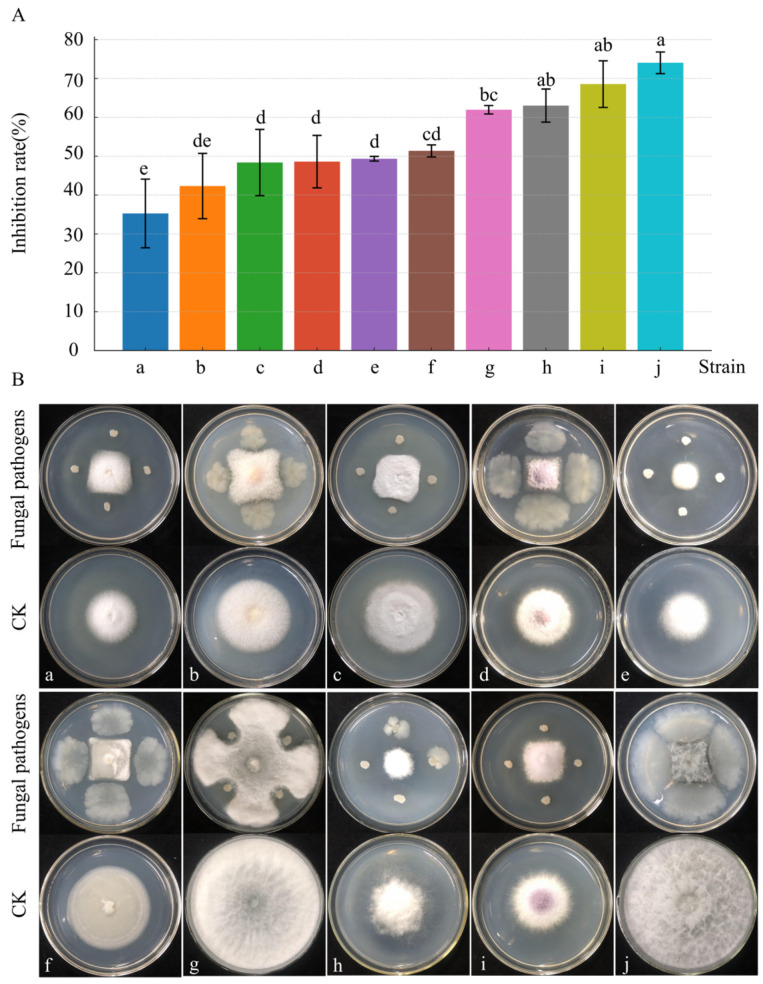
Antimicrobial spectrum of strain D39. (**A**) Histogram of the inhibitory effect of strain D39 on pathogenic fungi a–j. Different lowercase letters denote significant differences among treatments (Waller–Duncan test, *p* < 0.05). (**B**) Representative images of the inhibition effect of strain D39 on pathogenic fungi a–j. Pathogenic fungi a–j are, respectively: *F. solani*, *F. tricinctum*, *P. capsici*, *F. fujikuroi*, *A. phaeospermum*, *F. incarnatum–equiseti species complex*, *B. dothidea*, *A. rasikravindrii*, *F. oxysporum*, and *N. parvum*.

The inhibitory effects of strain D39 varied across 10 pathogenic fungi (Figure 4). After 4 days of dual-culture, the highest inhibition rate (74%) was observed against *N. parvum*, and the lowest (35%) against *F solani*. Four pathogenic fungi—*N. parvum*, *F. oxysporum*, *B. dothidea*, and *A. rasikravindrii*—showed inhibition rates above 60%. These findings indicate that strain D39 exhibits broad-spectrum antifungal activity and holds promise as an agricultural biocontrol agent.

#### 3.3.4. Detection of Functional Genes

**Figure 5:**
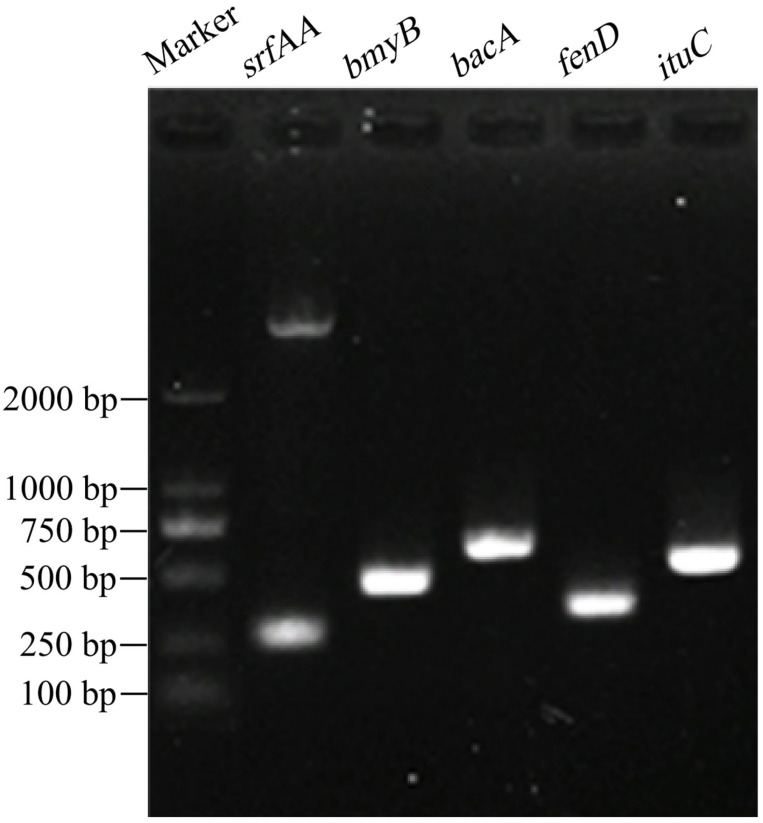
Detection of functional genes in *Bacillus amyloliquefaciens* D39.

PCR amplification was performed using five pairs of specific primers, and the products were examined by agarose gel electrophoresis, with all five primer pairs producing target bands (Figure 5). BLAST analysis revealed that the sequences of *srfAA*, *bmyB*, *bacA*, *fenD*, and *ituC* amplified from *B. amyloliquefaciens* D39 had nucleotide identities of 95.57%, 98.5%, 100%, 99.12%, and 98.95%, respectively, with corresponding genes in other *Bacillus species*. The gene fragment sizes were 177, 340, 461, 242, and 396 bp, respectively (Table 3), which are close to the sizes reported previously [44]. The genes *srfAA*, *ituC*, *fenD*, *bmyB*, and *bacA* were successfully amplified from strain D39, are involved in the biosynthesis of the antimicrobial compounds surfactin, iturin, fengycin, bacillomycin, and bacilysin, respectively, indicate the strong antimicrobial potential of *B. amyloliquefaciens* D39.

### 3.4. Pot Experiment for Efficacy Evaluation

**Figure 6:**
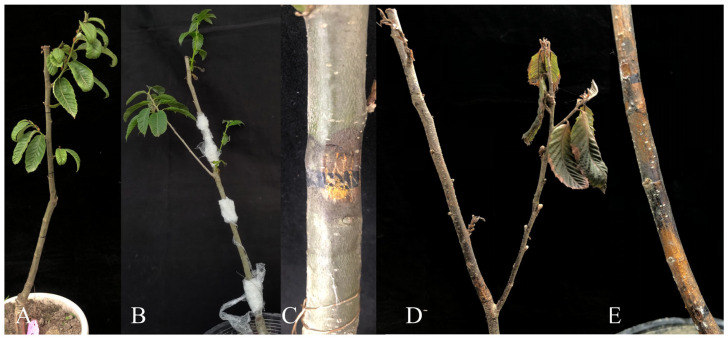
Control effect of *B. amyloliquefaciens* D39 fermentation broth on chestnut blight. (**A**) Healthy plants; (**B**,**C**) plants inoculated with the fermentation broth of strain D39; (**D**,**E**) control plants inoculated with distilled water.

The results of the pot experiment are shown in Figure 6 and Table 4. Before treatment, no significant differences were observed in disease incidence between the treatment and control groups (*p* > 0.05). On days 10 and 20 after treatment with the bacterial fermentation broth, the disease incidence increased in both groups. However, the disease incidence and disease severity index in the treatment group were significantly lower than those in the control group (*p* < 0.05), with control efficacy rates of 66.07% and 70.89%, respectively. Taken together, these results indicate that the fermentation broth of *B. amyloliquefaciens* D39 effectively inhibits the development of chestnut blight.

### 3.5. Characteristics of Antimicrobial Substances Produced by B. amyloliquefaciens D39

#### 3.5.1. Effect of Different Ammonium Sulfate Saturation Levels on Protein Precipitation

**Figure 7:**
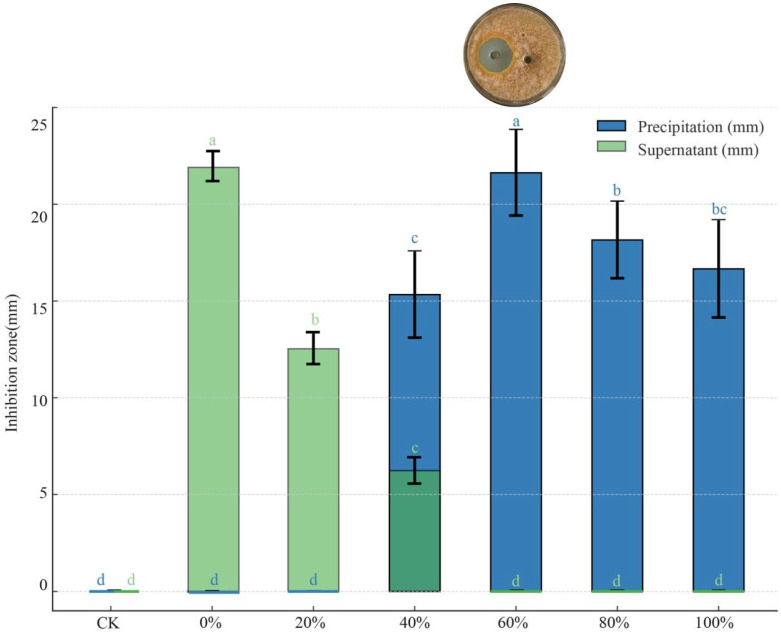
Inhibitory effect of precipitate and supernatant obtained by ammonium sulfate salting-out at different saturation levels against *C. parasitica*. Different lowercase letters denote significant differences among treatments (Waller–Duncan test, *p* < 0.05). The dark green bars indicate overlapping values of precipitation and supernatant at the corresponding saturation level.

The antimicrobial effects of the precipitates and supernatants obtained after ammonium sulfate fractionation at different saturation levels showed significant differences (*p* < 0.05). As the solution saturation increased, the antimicrobial effect of the precipitate initially intensified, reaching a peak inhibition zone diameter of 21.63 mm at 60% saturation, before subsequently declining (Figure 7). In contrast, the antimicrobial effect of the supernatant diminished as saturation increased, with no antimicrobial activity detected at 60% saturation or above.

The precipitation of antimicrobial substances from strain D39 after ammonium sulfate fractionation suggests that these substances may be proteinaceous in nature. The maximum amount of antimicrobial protein was precipitated at 60% saturation.

#### 3.5.2. Results of DEAE Sepharose Fast Flow and Sephadex G-75 Chromatography

**Figure 8:**
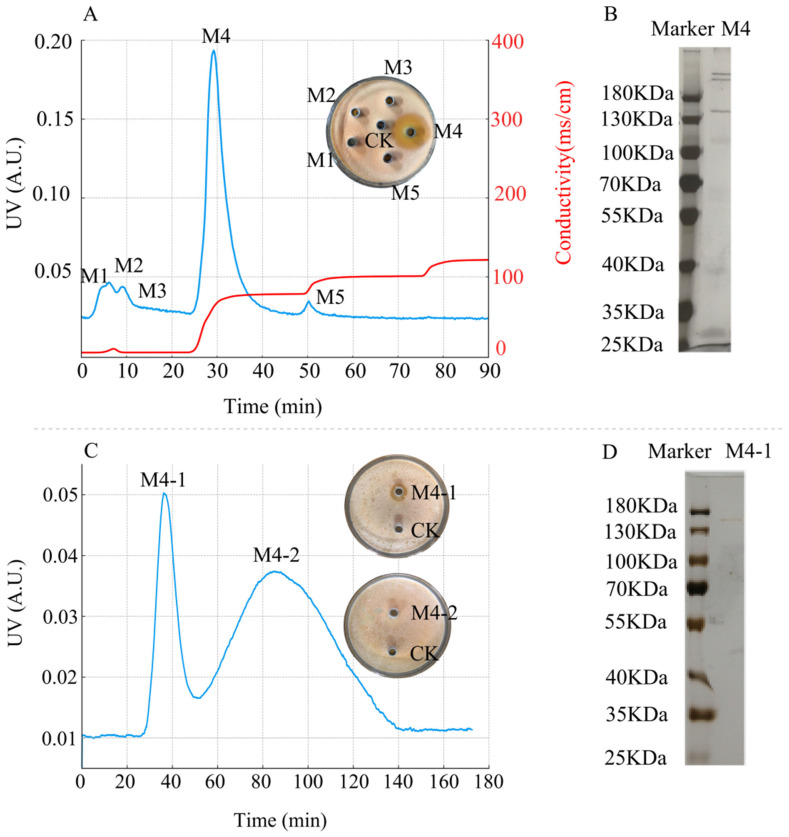
Purification of antimicrobial protein from strain D39. (**A**) DEAE Sepharose Fast Flow anion-exchange chromatography profiles and antimicrobial activity of the protein peaks. The blue line represents UV absorbance at 280 nm (left *Y*-axis), and the red line represents conductivity (right *Y*-axis). (**B**) SDS–PAGE analysis of protein peak M4. (**C**) Sephadex G-75 gel filtration chromatography profiles and antimicrobial activity of its protein peaks. (**D**) SDS–PAGE analysis of protein peak M4-1.

The crude protein precipitated obtained at 60% ammonium sulfate saturation was separated using a DEAE Sepharose Fast Flow chromatography column, resulting in five protein peaks, designated as M1–M5, with M4 exhibiting antimicrobial activity (Figure 8A). SDS–PAGE analysis revealed that protein peak M4 was a mixture of proteins (Figure 8B).

The further purification of protein M4 using a Sephadex G-75 gel filtration chromatography resulted in two protein peaks, designated as M4-1 and M4-2, with M4-1 displaying antimicrobial activity (Figure 8C). SDS–PAGE analysis revealed that protein peak M4-1 appeared as a single band (Figure 8D). The antimicrobial activity graphs for M4 and M4-1 indicated that the inhibition zone of M4-1 was smaller than that of M4. The smaller inhibition zone may be attributed to the loss of some antimicrobial proteins during Sephadex G-75 chromatography, leading to a decrease in protein concentration and content, thereby weakening the antimicrobial effect.

Using the Bradford method, the protein concentration of M4 was determined as 76.95 μg/mL, and that of M4-1 was 53.14 μg/mL, which supports the observed differences in their antimicrobial activity. Based on the standard curve of the logarithm of protein molecular weight versus relative mobility (*y* = −1.0591x + 2.3071, R^2^ = 0.9781, where *x* is the mobility and *y* is the logarithm of the molecular weight), the apparent molecular weight of the antimicrobial protein M4-1 was 145 kDa.

#### 3.5.3. RPLC-MS Identification and Analysis Results

**Figure 9:**
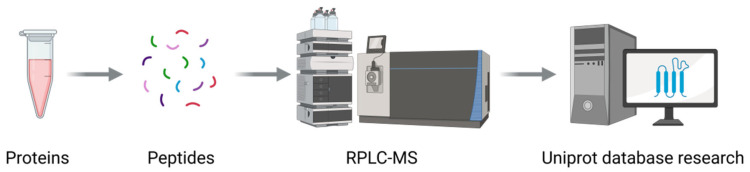
The experimental workflow for protein identification analysis.

Figure 9 shows the experimental workflow for protein identification analysis. The results from the RPLC-MS identification of the isolated and purified antimicrobial protein M4-1 from strain D39 were compared against the UniProt database (Table 5). According to the SDS–PAGE analysis, the target protein is located between 130 and 180 kDa. Two proteins, A0A6M9ZCG3 and A0A6M9ZHR5, conform to this range. However, the confidence score and the number of unique peptides of A0A6M9ZCG3 are both higher than those of A0A6M9ZHR5. Therefore, protein M4-1 was identified as belonging to the S8 family of serine peptidases, with a UniProt ID of A0A6M9ZCG3 and a confidence score of 158.94. This protein contains 1429 amino acids, with a theoretical molecular weight of 154.19 kDa, which is consistent with the apparent molecular weight (145 kDa) calculated from the SDS–PAGE analysis.

### 3.6. Protein Structure Analysis

The primary structure and physicochemical properties of the identified protein were analyzed using the ExPASy online tool. The protein has a theoretical isoelectric point of 6.03. The instability index was 25.6, indicating that the protein is relatively stable, and the average hydrophilicity was −0.602, suggesting that the protein is hydrophilic.

The secondary structure of the protein was predicted using PSIPRED (Figure S2) and consists of 21 α-helix segments (13.3%), 82 β-strand segments (33.17%), and random coils (53.53%).

**Figure 10:**
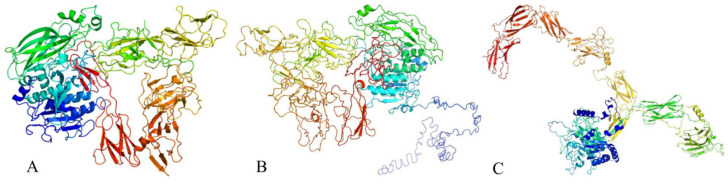
Prediction diagram of the tertiary structure of antimicrobial proteins. Predicted tertiary structure of the protein obtained using (**A**) Phyre2, (**B**) I-TASSER, and (**C**) trRosetta.

The tertiary structure of the protein was predicted using various bioinformatics tools. Phyre2 predicted the structure with 100% confidence and 74% coverage (Figure 10A), based on the template c6vjbA, achieving a TM-score of 0.1767. I-TASSER predicted the tertiary structure with a TM-score of 0.41 ± 0.14 (Figure 10B), while the prediction from trRosetta yielded a TM-score of 0.543 (Figure 10C)

## 4. Discussion

*B. amyloliquefaciens* has a broad antimicrobial spectrum and exhibits antagonistic activity against various pathogens, including gray mold, green mold in citrus, and bacterial fruit blotch [54,55,56,57]. Significantly, the *B. amyloliquefaciens* strain D39 inhibited the growth of the chestnut blight pathogen and exhibited varying inhibitory effects against more than ten different pathogenic fungi. The differential inhibitory effects likely arise from strain-specific antimicrobial strategies that dynamically modulate metabolite production (in both type and quantity) in response to pathogen challenges, with inter-strain activity variations. Mechanistic investigations revealed two predominant operational modes: (i) biosynthesis of bioactive metabolites (including lipopeptides such as surfactin, fengycin, and iturin, along with phenolic derivatives) that compromise cellular integrity through extracellular enzyme interference or membrane destabilization [58,59,60]; (ii) suppression of mycotoxin biosynthesis via critical gene inhibition [58]. However, different strains of *B. amyloliquefaciens* exhibit variable antagonistic efficacy against pathogenic fungi despite possessing equivalent numbers of lipopeptide-associated genes [61].

Furthermore, the growth curve and antimicrobial rate analysis of the *B. amyloliquefaciens* D39 revealed that both parameters peak at 24 h, which differs from other studies. Chen et al. [62] reported a growth peaked at 8 h, while Lee et al. [63] found that strain LN reached its maximum growth at 24 h, followed by a decline. In this study, although bacterial counts declined after 24 h, the inhibition rate remained stable. The differences between studies may be attributed to factors such as the initial inoculum size, culture volume, measurement intervals, and the test organisms used.

At the genetic level, *Bacillus* strains possessing a greater number of antimicrobial peptide biosynthesis genes, such as *srfAA*, *bacA*, and *fenD*, demonstrate more effective inhibitory activity against pathogens compared with other isolates [64]. Mora et al. [44] isolated 184 *Bacillus* strains, most of which contained 2–4 antimicrobial peptide genes, with 3.3% of the strains containing five genes, and none containing six genes. In addition, *Bacillus halotolerans* QTH8 was found to contain seven of these genes [65]. Strain D39 isolated in this study contains five antimicrobial peptide genes—*srfAA*, *ituC*, *fenD*, *bmyB*, and *bacA*—indicating its strong antimicrobial potential and high research value.

The pot experiment further validated the in vitro findings by demonstrating the biocontrol potential of B. amyloliquefaciens D39 under greenhouse conditions. The fermentation broth of strain D39 resulted in consistently lower disease incidence and severity compared to the control group. Notably, the disease control efficiency remained above 65% at both 10 and 20 days after treatment, suggesting that D39 not only suppresses early pathogen development but may also confer sustained protection. These findings imply that the biocontrol effect may arise from a combination of extracellular antifungal metabolites and possibly induced systemic resistance in the host. Such stable performance highlights the feasibility of developing D39-based bioformulations for practical chestnut blight management.

In terms of secondary metabolites, *B. amyloliquefaciens* produces various bioactive substances with antifungal, antibacterial, and nematicidal properties, including cyclic lipopeptides (e.g., fengycin, surfactins, bacillomycin D, and iturin A), polyketides (bacillaene, difficidin, and macrolactin), dipeptides (bacilysin), peptides (plantazolicin), and volatile organic compounds (e.g., 2,3-butanediol and acetoin) [66,67,68]. These compounds can be isolated, detected, and identified using various methods. For instance, Chen et al. [69]. usd semi-preparative high-performance liquid chromatography (HPLC) and electrospray ionization quadrupole time-of-flight mass spectrometry (ESI–Q-TOF MS)to isolate and identify iturin A as the major antifungal component active against *Botryosphaeria dothidea*. Huang et al. [70] applied silica gel, Sephadex LH-20, and HPLC purification techniques to isolate and purify the antibacterial compounds produced by *B. amyloliquefaciens* HR62, and subsequently identified these compounds, which inhibited the growth of *Ralstonia solanacearum*, as macrolactin A, 7-O-malonyl macrolactin A, and surfactin B using HPLC/ESI-MS.

To further investigate the active components, this study employed the pretreatment of proteins using ammonium sulfate precipitation, which facilitates protein aggregation by generating hydrophobic interactions between protein molecules, thereby reducing protein solubility and making it easier to separate them [71]. The antimicrobial protein from *B. amyloliquefaciens* D39 was isolated and purified using ammonium sulfate precipitation, DEAE Sepharose Fast Flow ion-exchange chromatography, and Sephadex G-75 gel filtration chromatography. Through RPLC–MS analysis, the protein was identified as belonging to the S8 family of serine peptidases (UniProt ID: A0A6M9ZCG3).

The S8 family, also known as the subtilisin family, is the second-largest group of serine protease. Owing to its industrial significance, subtilisin—the prototype of the S8 family—is one of the most extensively studied bacterial serine proteases. This study is the first to report the inhibitory effects of a serine peptidase of the S8 family against the chestnut blight pathogen, laying a theoretical foundation for the development of biocontrol agents against chestnut blight. Notably, S8 family serine proteases have demonstrated antifungal activities against various plant pathogens. For instance, in *Clonostachys chloroleuca*, the S8 serine protease CrKP43 interacts with MAP kinase Crmapk and is involved in mycoparasitism against *Sclerotinia sclerotiorum* and *F. oxysporum* [72]. In *Bacillus licheniformis* TG116, a serine protease exhibited antifungal activity against multiple pathogens, including *Phytophthora capsica*, *Rhizoctonia solani*, *Fusarium graminearum*, *F. oxysporum*, and *Botrytis cinerea* [73]. These examples underscore the potential of S8 family serine proteases as effective biocontrol agents against a broad spectrum of plant pathogens.

Given the importance of protein structure, structural analysis is crucial in practical applications such as modifying protein conformation or redesigning artificial proteins [74,75]. Currently, protein structures can be experimentally determined using X-ray crystallography and nuclear magnetic resonance spectroscopy [76,77]. However, these methods are costly and time-consuming, and the diversity of protein sequences poses significant challenges for experimental structure determination [78]. Alternatively, the primary structure of a protein—that is, the type and sequence of amino acids—determines its tertiary structure [79]. Based on this principle, computational protein structure prediction can rapidly provide relatively accurate protein structures at a lower cost compared with that of experimental methods. In this study, the tertiary structures of the isolated protein were predicted using three online tools. According to the TM-score values, Phyre2 had a relatively lower prediction accuracy, whereas trRosetta had the highest prediction accuracy. The predicted structures require further experimental validation. The significant differences in the predicted tertiary structures obtained in this study may be due to the following reasons: (1) proteins may exhibit multiple conformations under physiological conditions, and different prediction methods may favor different conformations, leading to different protein structures; (2) the three tools utilized different prediction principles, methods, databases, and data-processing approaches; and (3) variations in parameter settings and systematic errors could impact the accuracy of the predicted structures. Therefore, the accuracy of protein structure prediction requires further improvement.

## 5. Conclusions

In this study, the *B. amyloliquefaciens* strain D39 was isolated and identified from the rhizosphere soil of healthy chestnut trees. Strain D39 exhibited broad-spectrum antifungal activity, demonstrating varying degrees of inhibitory effects against *C. parasitica* and 10 other pathogenic fungi. In pot experiments, strain D39 was able to inhibit the growth of *C. parasitica*. Further analysis revealed that the antimicrobial protein secreted by the secretions of strain D39 exhibited potent biocontrol effects against *C. parasitica*. RPLC-MS identification indicated that this protein belongs to the S8 family of serine peptidases. Collectively, these findings suggest that the *B. amyloliquefaciens* strain D39 and its extracts have strong potential as biocontrol agents against *C. parasitica*, the causative agent of chestnut blight.

## Figures and Tables

**Table 1:** Antimicrobial effect of soil isolates in primary and secondary screening.

Strain Number	Primary Screening		Secondary Screening
	Inhibition Rate (%)	Inhibition Zone Width (cm)	Inhibition Zone Diameter (cm)
D18	75.41 ± 1.56 ^ef^	0.42 ± 0.14 ^f^	−
D22	81.32 ± 0.62 ^bcd^	1.08 ± 0.07 ^bc^	2.28 ± 0.18 ^ab^
D30	65.76 ± 0.62 ^g^	0.24 ± 0.06 ^fg^	−
D36	64.93 ± 2.89 ^g^	0.13 ± 0.01 ^g^	−
D37	73.23 ± 4.36 ^f^	0.34 ± 0.15 ^f^	−
D38	83.19 ± 0.62 ^abc^	1.08 ± 0.07 ^bc^	−
D39	85.06 ± 0.62 ^ab^	0.7 ± 0.13 ^e^	2.39 ± 0.07 ^a^
D40	84.13 ± 0.93 ^abc^	0.83 ± 0.07 ^de^	2.05 ± 0.04 ^b^
D41	82.15 ± 2.94 ^bcd^	1.06 ± 0.15 ^bc^	−
D42	80.39 ± 1.25 ^cd^	1 ± 0.09 ^cd^	2.1 ± 0.14 ^b^
D43	81.14 ± 1.97 ^bcd^	1.24 ± 0.1 ^b^	−
D45	78.00 ± 1.90 ^de^	1.24 ± 0.08 ^b^	2.13 ± 0.32 ^ab^
D52	78.69 ± 3.12 ^de^	1.01 ± 0.15 ^cd^	−
D55	87.45 ± 2.07 ^a^	1.72 ± 0.02 ^a^	−

Values are expressed as means ± standard deviation. “−” indicates no bacteriostatic effect; different lowercase letters denote significant differences among treatments (Waller–Duncan test, *p* < 0.05).

**Table 2:** Physiological and biochemical characterization of strain D39.

Test	Reaction
Starch hydrolysis	+
Aerobic test	+
Catalase reaction	+
Voges–Proskauer	+
Nitrate reduction reaction	+
Methyl red	−
Citrate utilization	−
Gelatin liquefaction	+
Phenylalanine deaminase	−
Gram-stain	+

Symbols “+” and “−” represent positive and negative reactions, respectively.

**Table 3:** BLAST sequence similarity of functional genes from *Bacillus amyloliquefaciens* D39.

Gene	Size (bp)	GenBank Accession Number	Best Matches in GenBank	Scientific Name	Query Cover	Percent Identity
*srfAA*	177	OM830959	CP053376	*B. amyloliquefaciens*	89%	95.57%
*bmyB*	340	OM830955	KP453869	*B. amyloliquefaciens*	97%	98.5%
*bacA*	461	OM830956	MG800648	*B. subtilis*	95%	100%
*fenD*	242	OM830957	KP453873	*B. amyloliquefaciens*	93%	99.12%
*ituC*	396	OM830958	KT781920	*B. subtilis*	95%	98.95%

**Table 4:** Control effect of *B. amyloliquefaciens* D39 fermentation broth on *C. parasitica*.

Time	Treatment	Bacterial Fermentation Broth,	CK	Significance Analysis
Before prevention and treatment	Incidence rate (%)	26.67 ± 3.33	27.78 ± 1.92	NS
Day 10	Incidence rate (%)	40.00 ± 3.33	88.89 ± 1.92	Treatment: * Time: *
	Disease index	21.11 ± 0.96	62.22 ± 2.54	
	Control effect (%)	66.07 ± 1.55	−	−
Day 20	Incidence rate (%)	58.89 ± 10.71	98.89 ± 1.92	Treatment: * Time: *
	Disease index	25.56 ± 3.85	87.78 ± 0.00	
	Control effect (%)	70.89 ± 4.38	−	−

Values are expressed as means ± standard deviation. NS, *p* > 0.05; *, *p* < 0.05.

**Table 5:** Detailed list of proteins identified via RPLC-MS and PEAKS software analysis.

No.	Accession	−10lgP	Cov. (%)	Unique Peptides	Avg. Mass	Description
1	A0A5C8IVR9	245.12	29	10	38,666	Aminopeptidase YhfE
2	A0A6M9ZGL4	197.99	10	5	86,631	Peptidase G2
3	A0A6M9ZD65	170.03	13	5	64,115	Gamma-glutamyltransferase
4	A0A1Y0XB26	168.71	19	4	39,375	Cellulase
5	A0A5C8IRJ3	162.51	21	7	45,709	Peptidase T
6	A0A6M9ZCG3	158.94	6	4	154,192	S8 family serine peptidase
7	A0A5C8IRC6	158.65	21	5	20,619	Spore coat protein GerQ
8	A0A5C8II39	134.65	35	6	27,110	Uncharacterized protein
9	A0A5C8IP87	120.05	11	4	50,053	Dihydrolipoyl dehydrogenase
10	A0A6M9ZDC1	115.73	19	6	48,236	S8 family peptidase
11	A0A5C8IRK7	111.76	5	3	77,011	Catalase
12	A0A6M9ZHR5	101.54	3	4	139,053	Nitrate reductase (quinone)
13	A0A6M9ZII5	81.53	14	2	17,975	Stress protein
14	Q9F9Q4	81.53	6	1	19,564	Stress protein
15	A0A5C8IUA2	72.75	21	4	17,600	Spore coat protein
16	A0A6M9ZCB4	72.24	3	1	49,735	Dihydrolipoyl dehydrogenase
17	A0A6M9ZGS6	66.97	10	2	42,518	N-acetylglucosamine-6-phosphate deacetylase
18	A0A5C8IVB4	64.26	11	5	54,544	Catalase
19	A0A1Y0X703	63.15	8	2	30,200	D-aminopeptidase
20	A0A5C8IK53	62.68	11	3	39,282	Glutamyl aminopeptidase
21	A0A6M9ZKZ3	60.73	5	1	19,298	Type 1 glutamine amidotransferase

Accession: UniProt database accession number of the protein; Cov. (Coverage, %): percentage of the protein sequence covered by identified peptides; −10lgP: negative base-10 logarithm of the p-value, representing identification confidence.; Unique Peptides: number of unique peptides identified for the protein; Avg. Mass: average mass of the identified protein; Description: detailed description of the protein.

## Data Availability

The original contributions presented in the study are included in the article/Supplementary Materials; further inquiries can be directed to the corresponding author.

## Supplementary Materials

The following supporting information can be downloaded at: https://www.mdpi.com/article/10.3390/microorganisms13061302/s1, Figure S1: Morphological characteristics and physiological and biochemical effects of *Bacillus amyloliquefaciens* strain D39; Figure S2. Protein secondary structure prediction results. Table S1: Primer sequence information; Table S2: Liquid chromatography parameters; Table S3: Mass spectrometry parameters; Table S4: Database search parameters.
