# Supplementary material for: Isolation of Bacillus amyloliquefaciens D39 and Identification of Its Antimicrobial Proteins Active Against Chestnut Blight
Source: Microorganisms. 2025 Jun 3;13(6):1302. doi: 10.3390/microorganisms13061302 (PMC12195296; doi:10.3390/microorganisms13061302)
Supplement: Supplementary file 1 [file microorganisms-13-01302-s001.zip › microorganisms-3598953-supplementary.pdf]

Supplementary Material

Isolation of *Bacillus amyloliquefaciens* D39 and Identification of its Antimicrobial Proteins Active Against Chestnut Blight

Supplementary Figures

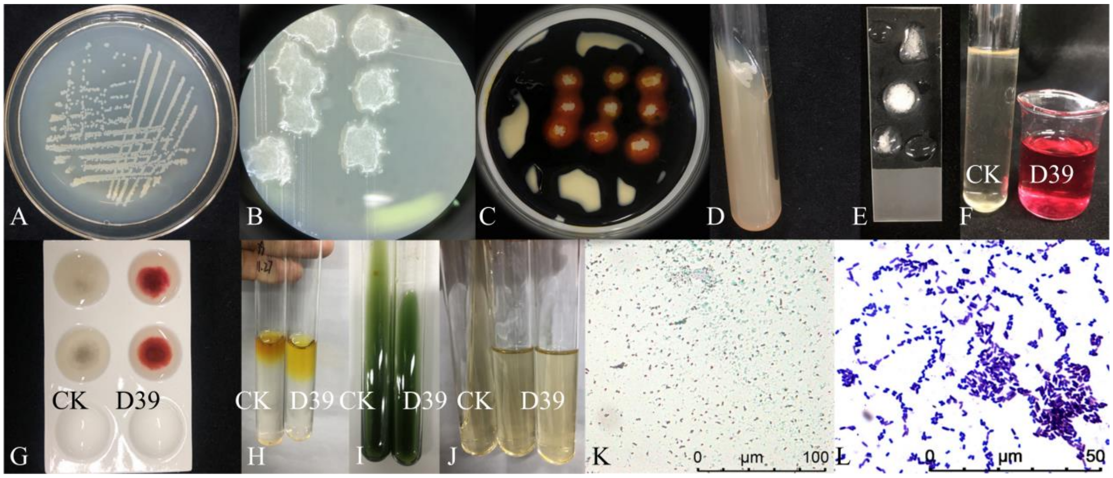

Figure S1. Morphological characteristics and physiological and biochemical effects of *Bacillus amyloliquefaciens* strain D39. **(A)** Colony morphology of strain D39 on LB agar; **(B)** colony morphology of strain D39 under a light microscope; **(C)** starch hydrolysis; **(D)** aerobic test; **(E)** contact enzyme reaction; **(F)** Voges–Proskauer test; **(G)** nitrate reduction reaction; **(H)** methyl red test; **(I)** citrate utilization test; **(J)** gelatin liquefaction; **(K)** spore staining; **(L)** Gram staining.

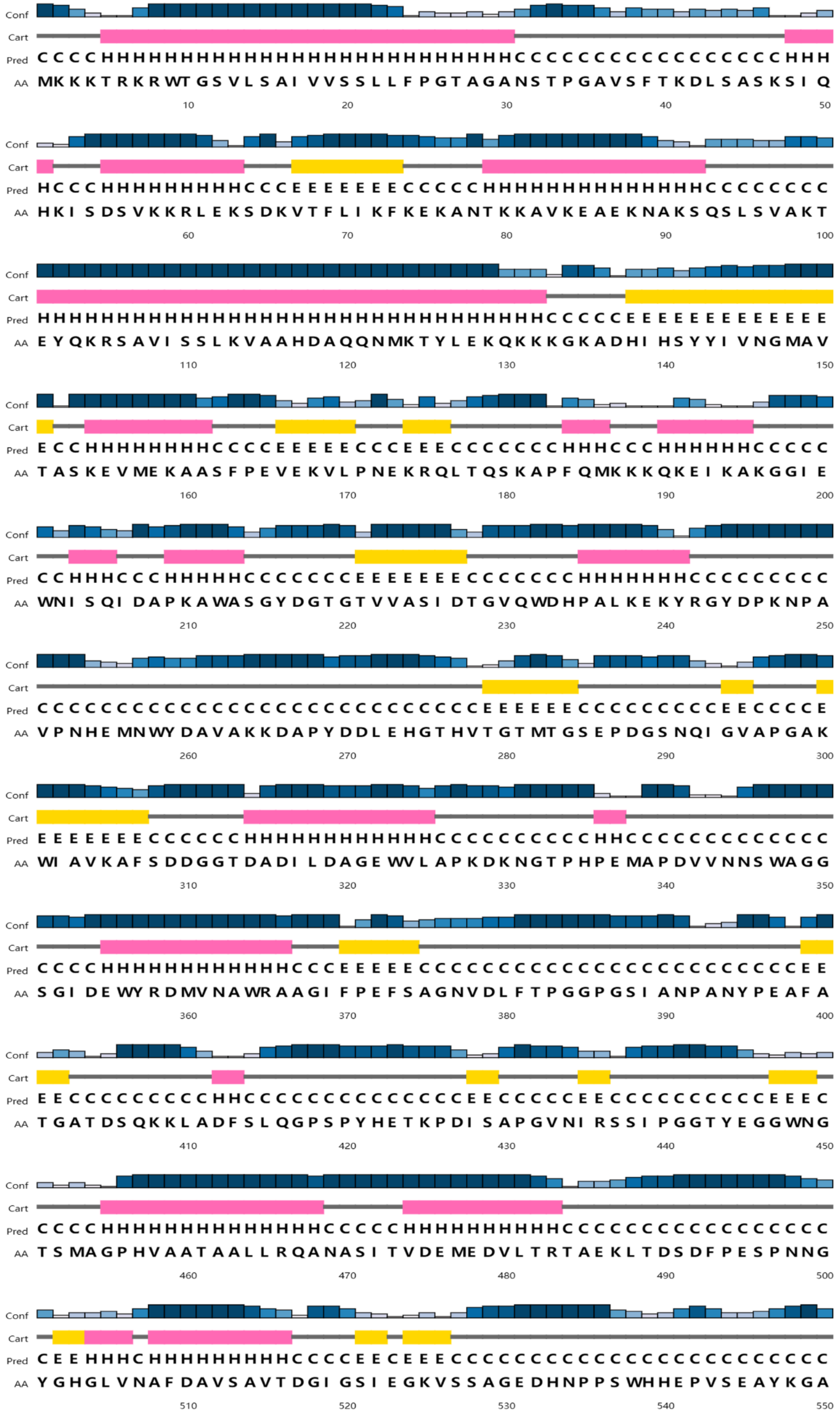
Conf
Cart
Pred CCCCHHHHHHHHHHHHHHHHHHHHHHHHHHCCCCCCCCCCCCCCCCCHHH
AA MKKKTRKRWTGSVLSAIVVSSLLFPGTAGANSTPGAVSFTKDLSASKSIQ
10 20 30 40 50
Conf
Cart
Pred HCCCHHHHHHHHHCCCEEEEEEECCCCCHHHHHHHHHHHHHHCCCCCCCC
AA HKISDSVKKRLEKSDKVTFLIKFKEKANTKKAVKEAEKNAKSQSLSVAKT
60 70 80 90 100
Conf
Cart
Pred HHHHHHHHHHHHHHHHHHHHHHHHHHHHHHHHCCCCCEEEEEEEEEEEEE
AA EYQKRSAVISSLKVAAHDAQQNMKTYLEKQKKKGKADHIHSYYIVNGMAV
110 120 130 140 150
Conf
Cart
Pred ECCHHHHHHHHCCCCEEEEECCCEEECCCCCCCHHHCCCHHHHHHCCCCC
AA TASKEVMEKAASFPEVEKVLPNEKRQLTQSKAPFQMKKKQKEIKAKGGIE
160 170 180 190 200
Conf
Cart
Pred CCHHHCCCHHHHHCCCCCCCEEEEEEECCCCCCCHHHHHHHCCCCCCCCC
AA WNISQIDAPKAWASGYDGTGTVVASIDTGVQWDHPALKEKYRGYDPKNPA
210 220 230 240 250
Conf
Cart
Pred CCCCCCCCCCCCCCCCCCCCCCCCCCCCEEEEEECCCCCCCCCEECCCCE
AA VPNHEMNWYDAVAKKDAPYDDLEHGTHVTGTMTGSEPDGSNQIGVAPGAK
260 270 280 290 300
Conf
Cart
Pred EEEEEEECCCCCCHHHHHHHHHHHHCCCCCCCCCCHHCCCCCCCCCCCCC
AA WIAVKAFSDDGGTDADILDAGEWVLAPKDKNGTPHPEMAPDVVNNSWAGG
310 320 330 340 350
Conf
Cart
Pred CCCCHHHHHHHHHHHHCCCEEEEECCCCCCCCCCCCCCCCCCCCCCCCEE
AA SGIDEWYRDMVNAWRAAGIFPEFSAGNVDLFTPGGPGSIANPANYPEAFA
360 370 380 390 400
Conf
Cart
Pred EECCCCCCCCCHHCCCCCCCCCCCCCCEECCCCCEECCCCCCCCCCEEEC
AA TGATDSQKKLADFSLQGPSPYHETKPDISAPGVNIRSSIPGGTYEGGWNG
410 420 430 440 450
Conf
Cart
Pred CCCCHHHHHHHHHHHHHHCCCCCHHHHHHHHHHCCCCCCCCCCCCCCCCC
AA TSMAGPHVAATAALLRQANASITVDEMEDVLTRTAEKLTDSDFPESPNNG
460 470 480 490 500
Conf
Cart
Pred CEEHHHCHHHHHHHHHCCCCEECEEECCCCCCCCCCCCCCCCCCCCCCCC
AA YGHGLVNAFDAVSAVTDGIGSIEGKVSSAGEDHNPPSWHHEPVSEAYKGA
510 520 530 540 550

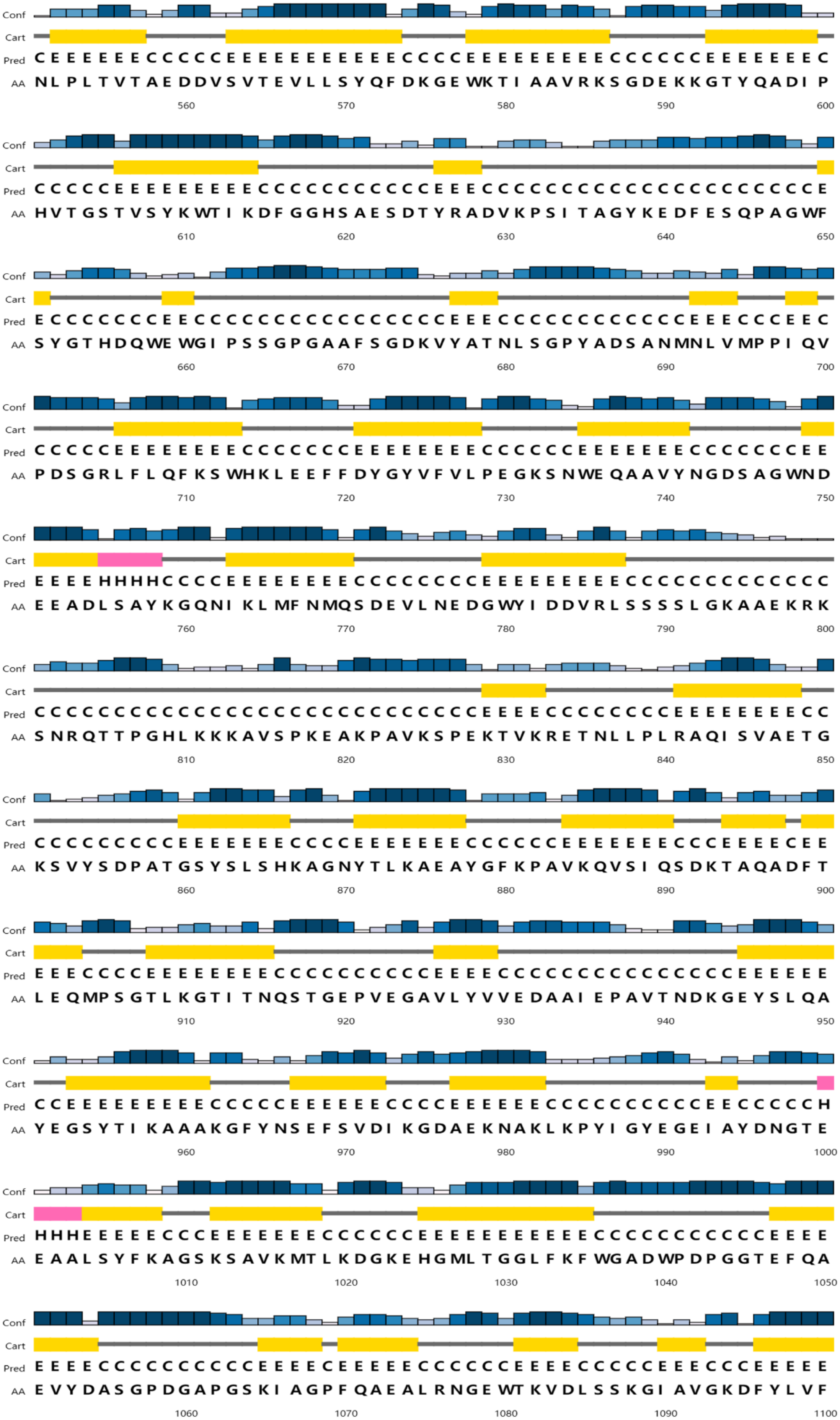
Conf
Cart
Pred C E E E E E E C C C C C E E E E E E E E E E E C C C C E E E E E E E E E C C C C C C E E E E E E E C
AA N L P L T V T A E D D V S V T E V L L S Y Q F D K G E W K T I A A V R K S G D E K K G T Y Q A D I P
560 570 580 590 600
Conf
Cart
Pred C C C C C E E E E E E E E E C C C C C C C C C C C E E E C C C C C C C C C C C C C C C C C C C C C E
AA H V T G S T V S Y K W T I K D F G G H S A E S D T Y R A D V K P S I T A G Y K E D F E S Q P A G W F
610 620 630 640 650
Conf
Cart
Pred E C C C C C C C E E C C C C C C C C C C C C C C C C E E E C C C C C C C C C C C C E E E C C C E E C
AA S Y G T H D Q W E W G I P S S G P G A A F S G D K V Y A T N L S G P Y A D S A N M N L V M P P I Q V
660 670 680 690 700
Conf
Cart
Pred C C C C C E E E E E E E E C C C C C C C E E E E E E E E C C C C C C E E E E E E E C C C C C C C E E
AA P D S G R L F L Q F K S W H K L E E F F D Y G Y V F V L P E G K S N W E Q A A V Y N G D S A G W N D
710 720 730 740 750
Conf
Cart
Pred E E E E H H H H C C C C E E E E E E E E C C C C C C C C E E E E E E E E E C C C C C C C C C C C C C
AA E E A D L S A Y K G Q N I K L M F N M Q S D E V L N E D G W Y I D D V R L S S S S L G K A A E K R K
760 770 780 790 800
Conf
Cart
Pred C C C C C C C C C C C C C C C C C C C C C C C C C C C C E E E E C C C C C C C C E E E E E E E E C C
AA S N R Q T T P G H L K K K A V S P K E A K P A V K S P E K T V K R E T N L L P L R A Q I S V A E T G
810 820 830 840 850
Conf
Cart
Pred C C C C C C C C C E E E E E E E C C C C E E E E E E E C C C C C C E E E E E E E C C C E E E E C E E
AA K S V Y S D P A T G S Y S L S H K A G N Y T L K A E A Y G F K P A V K Q V S I Q S D K T A Q A D F T
860 870 880 890 900
Conf
Cart
Pred E E E C C C C E E E E E E E E C C C C C C C C C C E E E E C C C C C C C C C C C C C C C E E E E E E
AA L E Q M P S G T L K G T I T N Q S T G E P V E G A V L Y V V E D A A I E P A V T N D K G E Y S L Q A
910 920 930 940 950
Conf
Cart
Pred C C E E E E E E E E E C C C C C E E E E E E C C C C E E E E E E C C C C C C C C C C E E C C C C C H
AA Y E G S Y T I K A A A K G F Y N S E F S V D I K G D A E K N A K L K P Y I G Y E G E I A Y D N G T E
960 970 980 990 1000
Conf
Cart
Pred H H H E E E E E C C C E E E E E E E C C C C C C E E E E E E E E E E E C C C C C C C C C C C E E E E
AA E A A L S Y F K A G S K S A V K M T L K D G K E H G M L T G G L F K F W G A D W P D P G G T E F Q A
1010 1020 1030 1040 1050
Conf
Cart
Pred E E E E C C C C C C C C C C E E E E C E E E E E C C C C C C E E E E C C C C C E E E C C C E E E E E
AA E V Y D A S G P D G A P G S K I A G P F Q A E A L R N G E W T K V D L S S K G I A V G K D F Y L V F
1060 1070 1080 1090 1100

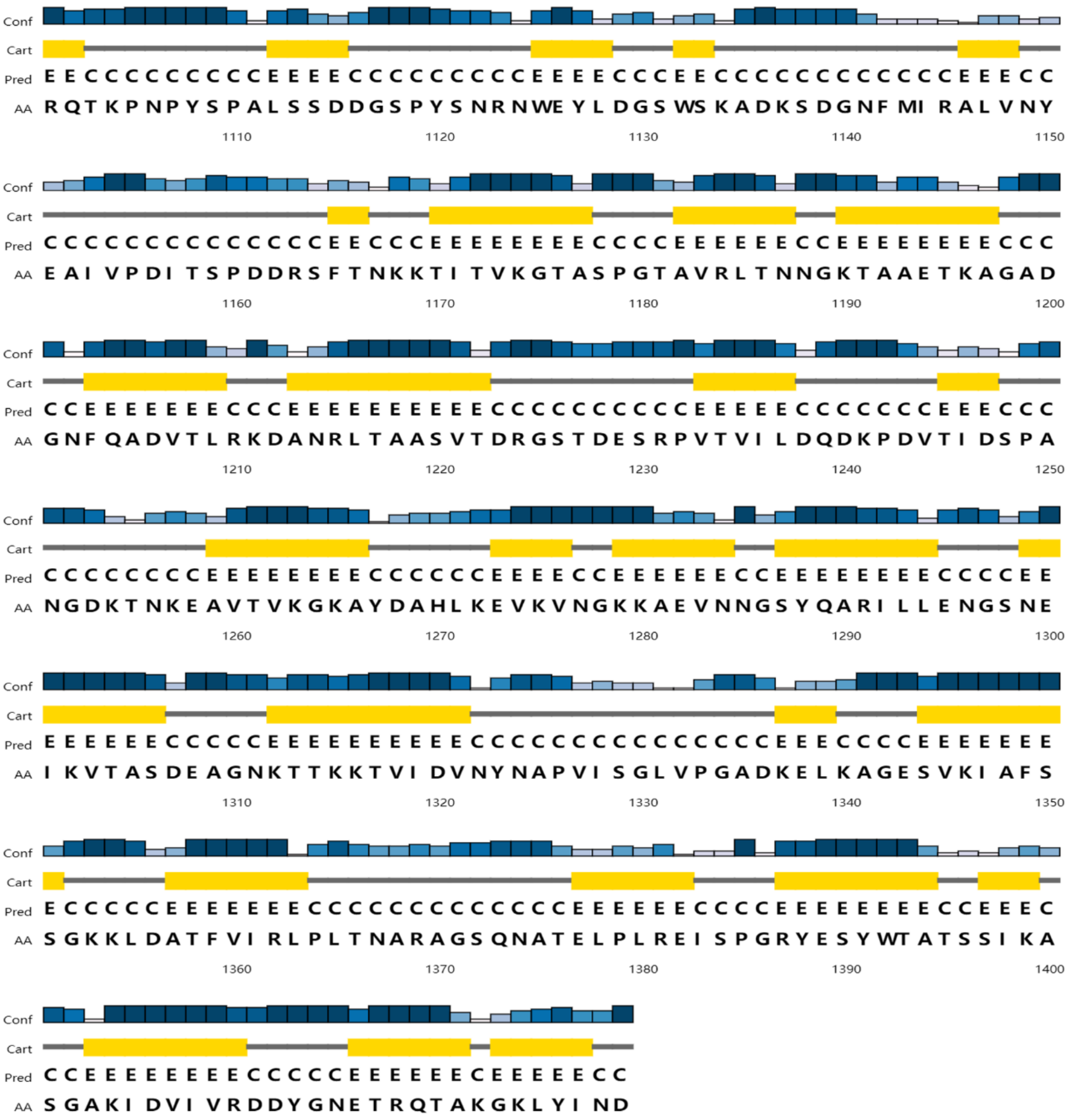

Figure S2. Protein secondary structure prediction results. Conf, confidence of prediction; Cart, Alpha-helix Beta-strand Random coil; Pred, Predicted secondary structure H, Alpha-helix; E, Beta-strand; C, Random coil AA, target sequence

Supplementary Tables

Table S1. Primer sequence information.

Gene	Expression product	Sequence (5′→3′)
srfAA	Surfactin	F: 5′-TCGGGACAGGAAGACATCAT-3′<br>R: 5′-CCACTCAAACGGATAATCCTGA-3′
ituC	Iturin	F: 5′-GGCTGCTGCAGATGCTTTAT-3′<br>R: 5′-TCGCAGATAATCGCAGTGAG-3′
fenD	Fengycin	F: 5′-GGCCCGTTCTCTAAATCCAT-3′<br>R: 5′-GTCATGCTGACGAGAGCAAA-3′
bmyB	Bacillomycin	F: 5′-GAATCCCGTTGTTCTCCAAA-3′<br>R: 5′-GCGGGTATTGAATGCTTGTT-3′
bacA	Bacilysin	F: 5′-CAGCTCATGGGAATGCTTTT-3′<br>R: 5′-CTCGGTCCTGAAGGGACAAG-3′

Table S2. Liquid chromatography parameters.

Time(min)	Flow Rate(nl/min)	A%	B%
0.00	400	97	3
3.00	400	97	3
7.00	400	92	8
46.00	400	68	32
51.00	400	56	44
56.00	400	1	99
60.00	400	1	99
60.10	300	97	3
70.00	400	97	3

Table S3. Mass spectrometry parameters.

	Item	Value
MS1	Resolution	120000
	AGC target	4e5
	Maximum IT	50 ms
	Scan range	350 to 1550 m/z
MS2	Resolution	30000
	AGC target	1e5
	Maximum IT	100 ms
	TopN	20
	NCE / stepped NCE	32

Table S4. Database search parameters.

Item	Value
Sequence Database Search	PEAKS
Protein Database	uniprot-proteome UP000502253.fasta
Enzyme	Trypsin
Max.Missed Cleavages per peptide	3
Precursor Mass Tolerance	10 ppm
Fragment Mass Tolerance	0.02 Da
Dynamic Modifications	Oxidation(M);Acetylation(N-term)
Static Modifications	Carbamidomethylation
